# Evaluation of cardiovascular biomarkers In HIV-infected patients switching to abacavir or tenofovir based therapy

**DOI:** 10.1186/1471-2334-11-267

**Published:** 2011-10-04

**Authors:** Thomas A Rasmussen, Martin Tolstrup, Jesper Melchjorsen, Christian A Frederiksen, Ulla S Nielsen, Bente L Langdahl, Lars Østergaard, Alex L Laursen

**Affiliations:** 1 Department of Infectious Diseases, Aarhus University Hospital, Skejby, Denmark; 2 Department of Anaesthesiology and Intensive Care, Aarhus University Hospital, Skejby, Denmark; 3 Department of Endocrinology and Internal Medicine, Aarhus University Hospital, Skejby, Denmark

**Keywords:** HIV, abacavir, tenofovir, cardiovascular disease

## Abstract

**Abstract:**

**Background**

Our objective was to evaluate and compare the effect of abacavir on levels of biomarkers associated with cardiovascular risk.

**Methods**

In an open-label randomized trial, HIV-infected patients were randomized 1:1 to switch from zidovudine/lamivudine to abacavir/lamivudine or tenofovir/emtricitabine. In the present analysis, we measured levels of interleukin-6 (IL-6), high-sensitivity C-reactive protein (hs-CRP), soluble intercellular adhesion molecule-1 (sICAM-1), soluble vascular adhesion molecule-1 (sVCAM-1), E-selectin, and myeloperoxidase (MPO) at baseline and 4, 12, and 48 weeks after randomization. D-dimer and fasting lipids were measured at baseline and weeks 12 and 48. Levels of biomarkers at all time points and changes from baseline were compared across study arms using Wilcoxon rank sum test.

**Results**

Of 40 included patients, 35 completed 48 weeks of randomized therapy and follow up. Levels of E-selectin (*P *= 0.004) and sVCAM-1 (*P *= 0.041) increased transiently from baseline to week 4 in the abacavir arm compared with the tenofovir arm, but no long-term increases were detected. We found no significant differences between study arms in the levels or changes in the levels of sICAM-1, MPO, d-dimer, IL-6, or hs-CRP. Levels of total cholesterol and high density lipoprotein (HDL) increased in the abacavir arm relative to the tenofovir arm, but no difference was found in total cholesterol/HDL ratio.

**Conclusion**

In patients randomized to abacavir-based HIV-treatment transient increases were seen in the plasma levels of E-selectin and sVCAM-1 compared with treatment with tenofovir, but no difference between study arms was found in other biomarkers associated with endothelial dysfunction, inflammation, or coagulation. The clinical significance of these findings is uncertain.

**Trial Regestration:**

Clinicaltrials.gov identifier: NCT00647244.

## Background

The association between abacavir (ABC) use and myocardial infarction (MI) has been heavily debated since the Data Collection on Adverse Events of Anti-HIV Drugs (DAD) Study Group reported an increased risk of MI in patients with current or recent ABC use [1]. Data from other cohort studies have provided conflicting results [2-7], whereas analyses from clinical trials generally do not lend support to a causal relationship between ABC and MI [8-11]. In brief, the association between ABC and risk of MI was also found in a Danish cohort study [4] and in the SMART study dataset [7]. On the other hand data from highly active anti-retroviral therapy (HAART) naïve patients in pooled GSK-sponsored trials [6], the AIDS Clinical Trial Group (ACTG) A5001 [2], the Veterans Health Administration's Clinical Case Registry [3], and a case-control study from The French Hospital Database on HIV [5] did not find a significant association between ABC and MI.

In stronger evidence level data from several randomized controlled trials (RCTs) comparing ABC with tenofovir disoproxil fumarate (TDF), no increase in rate of MI was detected among patients randomized to ABC-based compared to TDF-based therapy [9-11]. Only in the STEAL-study, in which patients assigned to the ABC arm had a higher prevalence of cardiovascular risk factors, was an increased rate of cardiovascular events with ABC use detected [8]. Most recently, in a trial level meta-analysis of RCTs in which ABC was randomized as part of a HAART regimen, no association between increased risk of MI and ABC was found [12].

However, of the RCTs mentioned above, only two have comprised patients with suppressed HIV-infection [8,9]. A feature of uncontrolled HIV replication is elevated levels of inflammatory markers that are associated with an increased risk of cardiovascular events [13-15], but initiation of HAART in treatment-naïve patients reduces the levels of these biomarkers [11]. Thus, some of the discrepancy between studies may have been caused by the confounding noise of uncontrolled HIV replication among HAART-naïve patients. Investigations of inflammatory biomarkers have not provided consistent evidence for increased coagulation, inflammation, or endothelial dysfunction during ABC treatment [7,11,16-20], but most studies have been limited by a lack of randomization and HLAB5701-screening, or included patients with uncontrolled HIV replication.

The Efficacy and Safety of Switching from Zidovudine to Tenofovir or Abacavir in HIV-infected Patients (SWAP) study was a randomized trial comparing switching from zidovudine (AZT) to ABC or TDF with parameters of renal function and bone metabolism as the pre-specified primary endpoints. However, the study provided an opportunity to perform a *post hoc *analysis to investigate biomarkers of cardiovascular risk as early as 4 weeks after treatment switch in patients with suppressed HIV-infection who were randomized to ABC or TDF treatment. We report here on the findings from the analyses of these cardiovascular biomarkers.

## Methods

### Patients

The SWAP study was an open-label, parallel-group, randomized clinical trial assessing the safety and efficacy of switching from AZT to ABC- or TDF-based therapy. Eligible patients had documented HIV-infection and, for at least 3 months, had been treated with HAART comprising AZT and had undetectable plasma HIV-RNA. A sample size of 90 participants was estimated as being required to detect differences in the primary outcome measures at a 5% significance level. Patients who had previously used ABC or TDF, had diabetes mellitus, untreated hypertension, or who were positive for the HLAB5701 allele upon screening were excluded. Informed consent was obtained before enrolment. At baseline, patients were stratified after use of protease inhibitors and randomized 1:1 to switch from AZT to ABC 600 mg or TDF 300 mg daily. If lamivudine (3TC) or emtricitabine (FTC) was given as part of HAART at inclusion, patients were randomized to a fixed-dose treatment with ABC/3TC 600/300 mg or TDF/FTC 300/200 mg daily. The study took place at the Department of Infectious Diseases, Aarhus University Hospital, Skejby, Denmark. Patients were followed up at 4, 8, 12, 24, and 48 weeks after randomization. A pre-planned follow up at 96 weeks was not performed because the study was discontinued in November 2009. The pre-specified primary endpoints were parameters of renal function, bone metabolism and bone mass density, lipodystrophy, and insulin resistance. Analysis of cardiovascular biomarkers was a sub-study that was added as an amendment when the suspicion of increased cardiovascular risk with ABC treatment was raised, but collection of plasma samples was part of the original protocol and did not require further selection of study subjects. Both the main and sub-study was approved by the Regional Research Ethics Committee, Central Denmark Region (Journal number: M-20070189; Eudract. number: 2007-004372-39) and conducted in accordance with *Good Clinical Practice*. Data on fasting lipids, fasting glucose, anthropometric information, age, sex, smoking habits, blood pressure, CD4+ cell counts, viral load, and duration of HIV-infection and HAART exposure were retrieved from the study database or patient records. Based on relevant data, Framingham score and estimated 10-year risk of cardiovascular events were calculated as described previously [21].

### Measurement of biomarkers

Plasma samples (EDTA- and citrate-treated) were stored at -80°C until analysis. To detect both early and late changes in inflammation and endothelial dysfunction markers, we analyzed levels of IL-6, high sensitivity (hs)-CRP, soluble intercellular adhesion molecule-1 (sICAM-1), soluble vascular adhesion molecule-1 (sVCAM-1), E-selectin, and myeloperoxidase (MPO) at baseline and at weeks 4, 12, and 48 after randomization in plasma. Citrate plasma was not collected at week 4 and, therefore, d-dimer levels were only measured at baseline and weeks 12 and 48. E-selectin, MPO, sICAM-1, and sVCAM-1 were measured in a multiplex assay on the Luminex platform as described by the manufacturer (Milliplex CVD Panel 1, Millipore, Copenhagen, Denmark). Inter- and intra-assay coefficient of variation (CV) ranged from 8.5-16.3% and 4.5-12.3%, respectively; lower limits of detection are 79 pg/mL for E-selectin, 16 pg/mL for sVCAM-1, 9 pg/mL for sICAM-1, and 7 pg/mL for MPO. Commercially available ELISA-based assays were used to determine levels of IL-6 (Invitrogen, CA, USA; intra-assay CV 4.71-8.33%, inter-assay CV 6.70-10.0%) and d-dimer (American Diagnostica Inc., CT, USA; intra-assay CV < 10%). The lower limits of detection were 4 μg/L for d-dimer and 0.104 pg/mL for IL-6. Hs-CRP levels were analyzed with a high-sensitivity in-house sandwich ELISA with a lower detection limit of 0.2 mg/L. All measurements were performed in duplicate. Samples with undetectable levels were assigned the value of the lower detection limit.

### Statistics

Baseline data were compared using χ^2^-test for categorical variables and two sample t-test or Wilcoxon rank sum test for continuous variables, depending on a skewed or Gaussian distribution of data, which was assessed through visual inspection of frequency histograms and normal probability plots. Median levels of biomarkers at baseline and at weeks 4, 12, and 48, as well as relative changes from baseline to weeks 4, 12, and 48 were compared across the 2 study arms by Wilcoxon rank sum test. Because the prognostic significance of hs-CRP for cardiovascular events is well established [22] and levels >1 mg/L predict for higher risk of adverse cardiovascular events [23], we compared the proportion of patients in each arm with hs-CRP > 1 mg/L at all time points by χ^2^-test in a *post hoc *analysis. Median levels of fasting lipids at baseline, week 12, and week 48, as well as relative changes from baseline to weeks 12 and 48, respectively, were compared between the 2 study arms by Wilcoxon rank sum test. Stata 10 was used for statistical analyses. *P*-values < 0.05 were considered statistically significant.

## Results

### Study group

**Figure 1:**
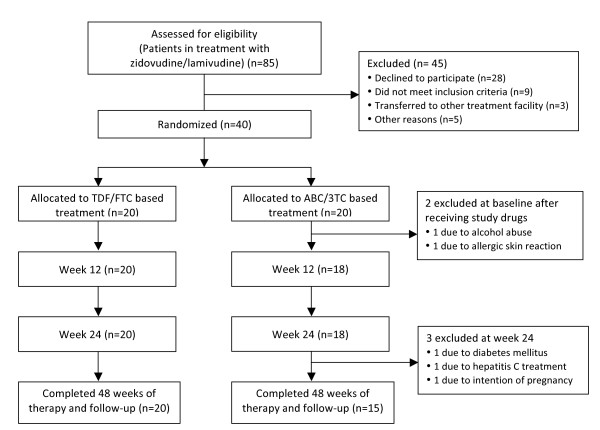
**Flow chart of inclusion and follow up**. No figure legends.

Because of uncertainty about ABC as a first-line treatment option, patient inclusion was suspended in January 2009 to await further confirmation on this issue. The study was finally terminated in August 2009. The flow of patient inclusion and follow-up is illustrated in Figure 1.

Forty patients were enrolled during the period June to December 2008, with 20 in each study arm. Two patients in the ABC/3TC arm were excluded shortly after randomization and contribute only to baseline data. One patient was excluded because of an allergic skin reaction upon starting ABC therapy (the patient was tested negative for the HLAB5701 allele), and one patient was excluded because of alcohol abuse and poor adherence to study drugs. Three patients in the ABC/3TC arm were excluded at week 12 for the following reasons: initiated treatment for hepatitis C, intention of pregnancy, and a history of diabetes mellitus, respectively. The latter patient had a history of diabetes prior to randomization and was listed as a screening failure. All 3 patients contributed data up to and including week 12. The remaining 35 study subjects all completed 48 weeks of therapy. One patient (TDF/FTC arm) did not provide the week-4 blood sample. One serious adverse event was reported during the study; this was a resolved episode of pyelonephritis in a patient with a history of recurrent urinary tract infections. Five grade 2 adverse advents were registered (4 in the ABC/3TC arm and 1 in the TDF/FTC arm), but none necessitated discontinuation of study drugs. No grade 3 or 4 adverse advents were reported.

Baseline characteristics of the patients are shown in Table 1. All patients received zidovudine (AZT) in combination with 3TC at inclusion, and were randomized to either ABC/3TC or TDF/FTC. The proportion of smokers and males was higher and duration of HAART exposure longer in the TDF/FTC arm. Patients in both arms were virologically suppressed throughout the study.

**Table 1:** Baseline characteristics

Parameter	ABC/3TC (n = 20)	TDF/FTC (n = 20)
Mean age (SD), years	50 (11.9)	46 (8.7)
Male sex, n (%)	11 (55.0)	14 (70.0)
Active smokers, n (%)	6 (30.0)	9 (45.0)
Mean weight (SD), kg	73.2 (12.1)	74.9 (16.3)
Mean BMI (SD), kg/m^2^	25.2 (2.1)	25.0 (4.3)
Mean systolic blood pressure (SD), mmHg	133 (21.9)	135 (15.1)
Mean diastolic blood pressure (SD), mmHg	85 (10.9)	84 (9.4)
Median total cholesterol (IQR), mmol/L	5.7 (5.3-6.7)	5.35 (4.8-6.0)
Median LDL cholesterol (IQR), mmol/L	3.8 (3.1-4.3)	3.2 (2.6-4.0)
Median HDL cholesterol (IQR), mmol/L	1.3 (1.1-1.9)	1.2 (1.0-1.7)
Median triglyceride (IQR), mmol/L	1.5 (1.2-2.0)	1.4 (1.0-2.0)
Mean fasting plasma glucose (SD), mmol/L	5.4 (0.52)	5.4 (0.38)
Zidovudine/lamivudine at baseline, n (%)	20 (100)	20 (100)
PI at baseline, n (%)	6 (30.0)	6 (30.0)
Efavirenz at baseline, n (%)	11 (55.0)	9 (45.0)
Nevirapine at baseline, n (%)	3 (15.0)	5 (25.0)
Mean CD4+ cell count (SD), cells/mm^2^,	567 (256)	540 (206)
Median duration of HAART exposure (IQR), months	86.5 (54.5-125)	106.5 (50.5-130)
Median time since HIV-diagnosis (IQR), months	127 (96.5-140.5)	122 (89-179)
Median Framingham score (IQR)	3 (0-6)	4 (1-7)
Median estimated 10-year cardiovascular risk a.m. Framingham (IQR), %	7 (3-10)	6 (3-11)

BMI = body mass index; PI = protease inhibitor; HAART = highly active antiretroviral therapy; HIV = human immunodeficiency virus.

Normally distributed data are presented as means with standard deviation (SD) in parenthesis. Skewed data are presented as medians with interquartile range (IQR) in parenthesis.

### Cardiovascular biomarkers

**Figure 2:**
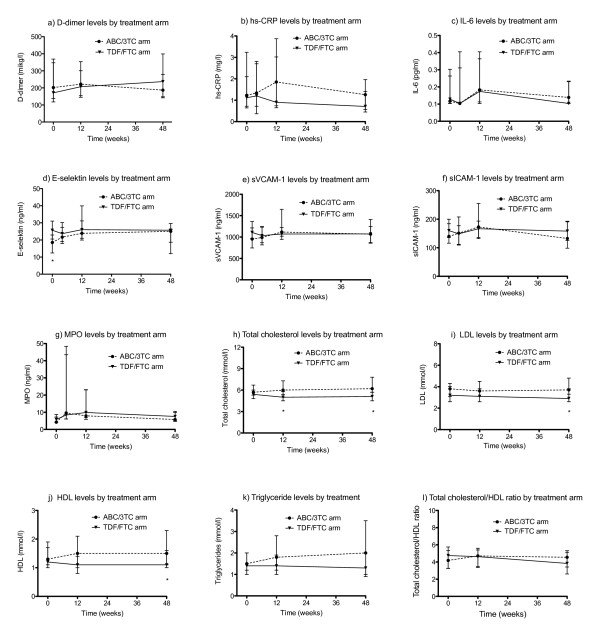
**Median levels of cardiovascular biomarkers and fasting lipids during study treatment according to study arm**. Hs-CRP = high sensitivity C-reactive protein; IL-6 = interleukin 6; sVCAM-1 = soluble vascular adhesion molecule-1; slCAM-1 = soluble intercellular adhesion molecule-1; MPO = myeloperoxidase; LDL = low density lipoprotein; HDL = high density lipoprotein. Whiskers indicate interquartile ranges. * P < 0.05 for ABC/3TC compared with TDF/FTC arm at the specific time point using Wilcoxon rank sum test.

The median levels of cardiovascular biomarkers at baseline and at weeks 4, 12, and 48 are shown in Figure 2. Relative changes from baseline to weeks 4, 12, and 48, respectively, are shown in Table 2. Levels of E-selectin were higher at baseline among patients in the TDF/FTC arm compared with the ABC/3TC arm (*P *= 0.014), but this difference levelled out at week 4 and did not reoccur at subsequent time points. Correspondingly, changes in levels of E-selectin from baseline to week 4 were significantly higher in the ABC/3TC arm compared with the TDF/FTC arm (*P *= 0.004). A similar pattern was observed with levels of sVCAM-1, which increased from baseline to week 4 in the ABC/3TC arm compared with the TDF/FTC arm (*P *= 0.041). For both markers, the increase was transient, and no significant changes in the level of E-selectin or sVAM-1 were detected from baseline to week 12 or 48, respectively. We performed analyses stratified for sex and age to examine whether the changes in E-selectin and sVCAM1 from baseline to week 4 could have been influenced by these variables, but we did not find any indications hereof.

**Table 2:** Relative change from baseline in the levels of cardiovascular biomarkers and fasting lipids

Biomarker (units)	ABC/3TC arm, % (IQR)	TDF/FTC arm, % (IQR)	*P *values
**D-dimer (μg/l)**			
Week 4	na	na	na
Week 12	9 (-10-44)	8 (-17-29)	0.770
Week 48	1 (-24-40)	22 (-9-47)	0.301
**hs-CRP (mg/l)**			
Week 4	41 (-32-82)	-27 (-50-59)	0.378
Week 12	32 (-36-106)	-9 (-46-44)	0.279
Week 48	23 (-44-120)	-27 (-59- [-]2)	0.110
**IL-6 (pg/ml)**			
Week 4	0 (-33-6)	0 (-22-88)	0.555
Week 12	0 (-15-58)	0 (-15-38)	0.883
Week 48	0 (-15-39)	0 (-25-29)	0.673
**E-selectin (ng/ml)**			
Week 4	14 (-1-31)	-7 (-28-3)	0.004
Week 12	19 (-2-56)	6 (-11-20)	0.152
Week 48	19 (-1-36)	6 (-11-21)	0.162
**sVCAM-1 (ng/ml)**			
Week 4	8 (-10-15)	-11 (-24-4)	0.041
Week 12	17 (-11-59)	6 (-19-17)	0.198
Week 48	2 (-12-16)	-3 (-15-12)	0.739
**sICAM-1 (ng/ml)**			
Week 4	-1 (-9-10)	-10 (-26-3)	0.061
Week 12	8 (-6-53)	9 (-8-21)	0.501
Week 48	0 (-18-15)	-3 (-12-13)	0.973
**MPO (ng/ml)**			
Week 4	77 (0-508)	31 (-9-821)	0.793
Week 12	51 (4-264)	67 (30-214)	0.726
Week 48	25 (-30-128)	11 (-78-71)	1.000
**Total cholesterol (mmol/l)**			
Week 12	4 (-2-13)	-3 (-11-0)	0.020
Week 48	11 (4-19)	-10 (-13-1)	0.002
**LDL (mmol/l)**			
Week 12	0 (-9-6)	-4 (-13-15)	0.640
Week 48	4 (-5-14)	-12 (-18-17)	0.117
**HDL (mmol/l)**			
Week 12	6 (-8-20)	0 (-19-0)	0.059
Week 48	23 (10-30)	0 (-9-9)	0.002
**Triglycerides (mmol/l)**			
Week 12	27 (-14-50)	-8 (-21-18)	0.065
Week 48	20 (-14-90)	0 (-25-12)	0.121
**Total cholesterol/HDL ratio**			
Week 12	2 (-15-16)	1 (-7-10)	0.872
Week 48	-6 (-16-3)	-4 (-20-8)	0.894

Hs-CRP = high sensitivity C-reactive protein; IL-6 = interleukin 6; sVCAM-1 = soluble vascular adhesion molecule-1; slCAM-1 = soluble intercellular adhesion molecule-1; MPO = myeloperoxidase; LDL = low density lipoprotein; HDL = high density lipoprotein; na = not available.

Relative change (%) from baseline in the levels of cardiovascular biomarkers and fasting lipids according to study arm for each biomarker; comparison across study arms performed by Wilcoxon rank sum test. Applying Bonferroni's correction for multiple testing would yield a significance level of 0.002 to be used in the statistical evaluation of all variables.

We observed no significant differences in the level of sICAM-1, MPO, d-dimer, IL-6, or hs-CRP between study arms at any time point. Also, changes in the level of sICAM-1, MPO, d-dimer, IL-6, or hs-CRP from baseline to weeks 4, 12, and 48, respectively, were not significantly different between study arms.

**Figure 3:**
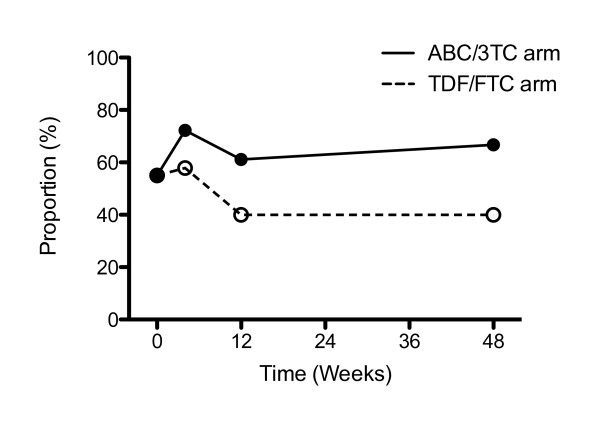
**Proportion with hs-CRP > 1 mg/L according to study arm**. The proportion of patients with hs-CRP > 1 mg/L is shown for each treatment arm at the various time points. At each time point, the two study arms are compared using χ^2^;-test; corresponding *P*-values are indicated above the graphs. Hs-CRP = high sensitivity C-reactive protein.

Levels of hs-CRP increased modestly in the ABC/3TC arm compared with the TDF/FTC the arm, but the change was below the level of significance. Accordingly, the proportion of patients with hs-CRP > 1 mg/L in the TDF/FTC arm decreased from baseline to week 12 and remained stable thereafter, whereas the opposite was true for the ABC/3TC arm, but the difference was non-significant (Figure 3).

### Fasting lipids

Median levels of fasting lipids at baseline and at weeks 12 and 48 are shown in Figure 2. Relative changes from baseline to weeks 12 and 48, respectively, are shown in Table 2. At baseline, there was a trend towards higher total cholesterol in the ABC/3TC arm compared with the TDF/FTC arm. This difference became statistically significant at subsequent time points and, accordingly, we detected significant increases in total cholesterol with ABC/3TC treatment compared with TDF/FTC treatment (*P *= 0.020 and *P *= 0.002 for relative changes at week 12 and 48, respectively). However, levels of high-density lipoprotein (HDL) also increased in the ABC/3TC arm compared with the TDF/FTC arm, and reached statistical significance by week 48 (*P *= 0.002 for relative change). Consequently, no difference in the total cholesterol/HDL ratio was found between study arms. Levels of low-density lipoprotein (LDL) were lower in the TDF/FTC arm throughout the study, but the difference between the two groups was only significant at 48 weeks. There was no difference between study arms with respect to the relative changes in LDL levels.

## Discussion

In this sub-study of the SWAP randomized clinical trial, the introduction of fixed-dose treatment with ABC/3TC lead to increases in plasma levels of E-selectin and sVCAM-1 compared with fixed-dose treatment with TDF/FTC, but the effect was short term and could not be detected after 12 and 48 weeks of follow up. No significant changes in the levels of sICAM-1, MPO, hs-CRP, or d-dimer were found.

As observed in previous randomized trials [8-10], we detected an increase in the level of total cholesterol and HDL in the ABC/3TC arm relative to the TDF/FTC arm, but there was no difference between total cholesterol/HDL ratios. It is therefore likely that the observed movement in fasting lipids will not significantly affect the 10-year cardiovascular risk, in line with what was seen in the STEAL study [8].

The strength of this study is its randomized design and that no further selection of participants was necessary, eliminating major sources of bias and confounding. Furthermore, the entry criteria required that patients be negative for the HLAB5701 allele, had not previously been exposed to ABC or TDF, and had been virologically suppressed for at least 3 months prior to randomization.

Other longitudinal studies did not find consistent patterns of increasing levels of cardiovascular biomarkers with ABC treatment. In a *post hoc *exploratory analysis of the HEAT study, changes in levels of CRP, sVCAM-1, and sICAM-1 were not significantly different among patients randomized to ABC/3TC or TDF/FTC [11]. It is noteworthy that participants in the HEAT study were HAART naïve at randomization and may not be comparable to the virologically suppressed patients in our study owing to the effect of uncontrolled HIV-replication on biomarker levels. The same applies for a recently published non-randomized study among viremic patients initiating abacavir or tenofovir, where no difference in changes of cardiovascular biomarkers, including early 4-weeks measurements, between treatment groups was found [24]. Similarly, a sub-study of the BIOCOMBO trial did not detect differences in markers of inflammation, endothelial dysfunction, hypercoagulability, or insulin resistance after treatment was switched to an ABC/3TC- or TDF/FTC-based regimen [18]. The STEAL study is particularly interesting because patients who switched to treatment with ABC/3TC, as compared to TDF/FTC, had a higher rate of cardiovascular events during follow up, but also a higher proportion of smokers and higher Framingham scores at baseline [8]. Nevertheless, an exploratory analysis did not show any consistent association between ABC/3TC and circulating levels of cardiovascular or inflammatory biomarkers, although a trend towards higher ICAM-1 levels in the ABC/3TC arm was observed [20].

ICAM-1 is the endothelial ligand for macrophage-1 antigen (Mac-1) that is expressed on the surface of leukocytes and mononuclear cells. Accumulation of these cells in the vessel wall is a hallmark of atherosclerosis and acute coronary syndrome, and is mediated by the interaction between adhesion molecules on endothelial and circulating cells. In an experimental system, Pablo et al. recently demonstrated that ABC but not AZT or 3TC induced significant and dose-dependent increases in rolling flux and adhesion in the leukocyte-endothelium interaction [25]; these effects were blocked by antibodies against ICAM-1 or Mac-1. Furthermore, treatment of neutrophils and monocytes with ABC increased the expression of Mac-1, whereas no increases in ICAM-1, VCAM-1, or E-selectin were seen following the treatment of endothelial cells [25]. Altogether, these results suggest that ABC treatment could be linked to increased expression of Mac-1 rather than endothelial adhesion molecules, which would explain the paucity of positive results in studies of the latter.

We found a transient effect of abacavir treatment on some, but not all of the investigated cardiovascular biomarkers. The main contribution of our analysis is the early evaluation of these at week 4; in other randomized trials such assessment was not done until week 12 [20] or 48 [11,18]. However, whether a temporary increase in E-selectin and sVCAM-1 may translate into an increased risk of myocardial infarction within such a short time frame seems unlikely, and the clinical significance of this finding, if any, is uncertain. In addition, for both E-selectin and sVCAM-1, baseline levels were higher in the TDF/FTC arm than in the ABC/3TC arm, though only significantly so for E-selectin. Hence, we cannot rule out that the statistical phenomenon of regression towards the mean is contributing to the relative increase of these two biomarkers in the ABC/3TC arm compared with the TDF/FTC arm from baseline to week 4.

Both E-selectin and sVCAM-1 are associated with atherosclerosis, risk of cardiovascular events, and risk of death in patients with known cardiovascular disease [26-29] even though these associations have not yet been proven in HIV-infected patients. The association of ABC use and risk of MI in the DAD study data was no longer present 6 months after stopping ABC [1], but the temporal behaviour of the risk within this period is unknown. Our data suggest that the effect on vascular inflammation is fast acting, short-lived, and that no continuous pro-inflammatory effect is present. This could be relevant when considering switching treatment from ABC in patients with low cardiovascular risk who have been on ABC for several years. Such patients may not benefit from a therapy change with respect to cardiovascular risk.

Hs-CRP is a well-established marker of risk prediction of coronary events [14,15,23] and contains prognostic information with a cut-off as low as 1 mg/L. In a *post hoc *analysis, we tested for differences in the proportion of patients with hs-CRP above this limit. However, even though the proportion of patients with hs-CRP > 1 mg/L, as well as overall levels of hs-CRP, seemed to decrease with TDF/FTC treatment as compared with ABC/3TC treatment, the differences were not significant. Because of the low prevalence of detectable plasma HIV-RNA in our samples, any decrease in inflammatory markers is unlikely to be caused by improved virological control. We have recently shown that TDF down-regulates production of pro-inflammatory cytokines following stimulation of monocytes with lipopolysaccheride [30], and we speculate that TDF may have inherent anti-inflammatory properties that affect both upstream and downstream inflammatory markers to a modest degree.

Our study is limited by the small number of patients, which reduces the statistical power to detect differences between groups. Furthermore, the large number of comparisons made increases the likelihood that significant findings could have arisen by chance alone. However, the observed transient increases in biologically related endothelial biomarkers, including a tendency towards a similar transient increase in sICAM-1, speak against this possibility. All 5 participants who were excluded from the study (2 after baseline and 3 after week 12 measurements) were in the ABC/3TC arm, introducing a potential source of bias. However, in only one of these patients, who had pre-existing diabetes, was the reason for exclusion related to metabolic or cardiovascular causes. This was a sub-study that was not planned in the original protocol, but collection of plasma samples and lipid measurements were already established, and further selection of patients was not necessary. In the case of IL-6 measurements, 55% of samples had undetectable levels and were assigned the value of the lower detection limit to allow for statistical analyses; these assigned values may not reflect the true IL-6 concentration.

## Conclusion

In conclusion, levels of total cholesterol and high-density lipoprotein (HDL) increased relatively during treatment with ABC/3TC compared with TDF/FTC, but no difference was found in total cholesterol/HDL ratio. We observed transient increases in the plasma levels of E-selectin and sVCAM-1 in patients randomized to ABC/3TC compared with TDF/FTC, but no difference was found in other biomarkers associated with endothelial dysfunction, inflammation, or coagulation. The clinical significance of these findings is uncertain.

## Competing interests

Lars Østergaard has received consultancy and speaker's fee from: Abbott, MSD, Pfizer, Bristol-Meyer Squibb, GlaxoSmithKline, ViiV Healthcare, Gilead, and Tibotec. Alex L Laursen has served on the advisory board for GlaxoSmithKline, Gilead, and Janssen. GlaxoSmithKline supported this study with a grant of DKR 100.000.

## Authors' contributions

TAR performed collection, analysis, and interpretation of data and drafted manuscript for publication. MT participated in the design of the study, coordinated laboratory analyses, and participated in the analysis and interpretation of data. JM set up and carried out in-house hs-CRP measurements, coordinated cardiovascular biomarker measurements and assisted in data analysis. CAF and USN contributed to the design of the study, performed participant inclusion and contributed substantially to acquisition of data. BLL participated in the design of the study and data interpretation. LØ participated in the design of study, data analysis, data interpretation, and helped to draft the manuscript. ALL conceived of the study, participated in its design and coordination, helped with data interpretation and drafting of the manuscript. All authors read and approved the final manuscript.

## Pre-publication history

The pre-publication history for this paper can be accessed here:

http://www.biomedcentral.com/1471-2334/11/267/prepub
